# Supplementary material for: Improving the detection and management of common non-communicable diseases in adults in rural Sidama National Regional State, Ethiopia: Study protocol
Source: PLoS One. 2026 Apr 20;21(4):e0302667. doi: 10.1371/journal.pone.0302667 (PMC13094975; doi:10.1371/journal.pone.0302667)
Supplement: Attachment — Submitted filename: Response to Reviewers.docx [file pone.0302667.s001.docx]

**Responses to the editor and the reviewer**

| **Manuscript ID:** | PONE-D-24-13998 |
| --- | --- |
| **Manuscript title (submission 1)** | Improving the detection and management of non-communicable diseases among the adult population of the catchment areas of a rural primary healthcare unit in Sidama National Regional State, Ethiopia: A study protocol |

# **Academic Editor’s comments**

**Comment 1**: “Please revise your manuscript in terms of general English.”

**Responses**: Thank you for your feedback. We have revised the language of the manuscript to improve clarity and readability. We utilized Microsoft Copilot and Grammarly to polish the language, reviewing and refining each paragraph to ensure the highest standard of English.

**Comment 2**: “A revised version of your manuscript that takes into account the comments of the referee(s) will be reconsidered for publication.”

**Responses**: We appreciate the valuable feedback provided by the referees. We have carefully revised the manuscript to address each comment and suggestion raised. Detailed responses to each referee's comments, along with a summary of the changes made to the manuscript, are provided below.

**Comment 3**: “Please ensure that your decision is justified on PLOS ONE’s publication criteria and not, for example, on novelty or perceived impact.”

**Responses**: We appreciate this comment. We have revised the resubmission to ensure that our protocol manuscript aligns strictly with PLOS ONE's publication criteria, which emphasize scientific rigor, ethical conduct, and methodological soundness. We have reviewed our study's objectives and methods to demonstrate their adherence to these criteria. We hope that these revisions address your concerns and look forward to your favorable consideration.

**Comment 4**:” Please include the following items when submitting your revised manuscript:

- “A rebuttal letter that responds to each point raised by the academic editor and reviewer(s). You should upload this letter as a separate file labeled **'Response to Reviewers'**.
- “A marked-up copy of your manuscript that highlights changes made to the original version. You should upload this as a separate file labeled **'Revised Manuscript with Track Changes'**.
- “An unmarked version of your revised paper without tracked changes. You should upload this as a separate file labeled **'Manuscript'**.”

**Responses**: Thank you for your guidance. As requested, we have submitted the three items: this rebuttal letter, the track changed manuscript, and the manuscript with accepted changes (unmarked) according to your recommendations.

**Comment 5**: “If you would like to make changes to your financial disclosure, please include your updated statement in your cover letter. “

**Responses**: We appreciate the suggestion. We have not made changes to our financial disclosure; thus, we have not updated the cover letter.

**Comment 6 (journal requirements):** When submitting your revision, we need you to address these additional requirements.

1. Please ensure that your manuscript meets PLOS ONE's style requirements, including those for file naming. The PLOS ONE style template can be found at <https://journals.plos.org/plosone/s/file?id=wjVg/PLOSOne_formatting_sample_main_body.pdf> and <https://journals.plos.org/plosone/s/file?id=ba62/PLOSOne_formatting_sample_title_authors_affiliations.pdf>
2. Please include your full ethics statement in the ‘Methods’ section of your manuscript file. In your statement, please include the full name of the IRB or ethics committee who approved or waived your study, as well as whether or not you obtained informed written or verbal consent. If consent was waived for your study, please include this information in your statement as well.

**Responses**: Thanks for letting us know. We have double-checked the revised manuscript to ensure it adheres to PLOS ONE's style guidelines, including file naming conventions. We've also referenced the style templates you provided.

Regarding ethical clearance, we have included an ethics statement in the Methods section (lines 517-544 in the revised manuscript). This statement outlines that ethical approval was obtained from the Institutional Review Board (IRB) of the College of Medicine and Health Sciences, Hawassa University (Ref. No: IRB/342/15). As the original protocol was modified, a revised ethical clearance was sought and approved by the Board (Ref. No: IRB/011/16). We also confirmed that participants were informed about the study's purpose and provided written consent, except for the quantitative data in Objective 5

# **Additional Editor’s comments**

**Comment 1**: “Please answer all the comments raised by the referee.”

**Responses**: Thank you for your guidance. We have thoroughly addressed each comment raised by the referee. Our detailed responses are included in this rebuttal letter, along with references to the revised manuscript.

**Comment 2**: “The manuscript needs amendments in terms of general English.”

**Responses**: Thank you very much for this comment. We have revised the general English in the updated submission as outlined in the Response to the Editor’s Comment 1.

# **Reviewer’s comments**

**Comment 1:** “Does the manuscript provide a valid rationale for the proposed study, with clearly identified and justified research questions?

“The research question outlined is expected to address a valid academic problem or topic and contribute to the base of knowledge in the field.

“Reviewer #1: Partly”

**Responses**: Thank you for your feedback regarding the rationale and research questions of our manuscript. We understand the importance of clearly identifying and justifying the research questions to address a valid academic problem and contribute to the field's base of knowledge.

In response to your comment, we have revised the manuscript to provide a more detailed rationale for the proposed study. We have also ensured that the research questions are more explicitly identified and justified, highlighting their relevance to addressing a significant academic issue. Specifically, we elaborated on the background and context of the study to better articulate the rationale. We also refined the research objectives in the revised submission.

We hope these revisions address the reviewer’s concerns. Thank you for helping us improve the manuscript.

**Comment 2**: “Is the protocol technically sound and planned in a manner that will lead to a meaningful outcome and allow testing the stated hypotheses?

“The manuscript should describe the methods in sufficient detail to prevent undisclosed flexibility in the experimental procedure or analysis pipeline, including sufficient outcome-neutral conditions (e.g. necessary controls, absence of floor or ceiling effects) to test the proposed hypotheses and a statistical power analysis where applicable. As there may be aspects of the methodology and analysis which can only be refined once the work is undertaken, authors should outline potential assumptions and explicitly describe what aspects of the proposed analyses, if any, are exploratory.

“Reviewer #1: Partly”

**Responses**: We appreciate your thoughtful feedback regarding the technical soundness of our protocol, as well as the comprehensive overview of our methods. We have revised the manuscript to address your concerns, ensuring that the methods are described with sufficient detail. Specific modifications to various elements of the methods are outlined in the "Review Comments to the Author" section below.

**Comment 3**: “Is the methodology feasible and described in sufficient detail to allow the work to be replicable?

“Descriptions of methods and materials in the protocol should be reported in sufficient detail for another researcher to reproduce all experiments and analyses. The protocol should describe the appropriate controls, sample size calculations, and replication needed to ensure that the data are robust and reproducible.

“Reviewer #1: Yes”

**Responses**: Thank you for your positive evaluation of the feasibility of our methodology and its detailed description.

**Comments 4**: Have the authors described where all data underlying the findings will be made available when the study is complete?

“The [PLOS Data policy](https://journals.plos.org/plosone/s/materials-and-software-sharing) requires authors to make all data underlying the findings described in their manuscript fully available without restriction, with rare exception, at the time of publication. The data should be provided as part of the manuscript or its supporting information, or deposited to a public repository. For example, in addition to summary statistics, the data points behind means, medians and variance measures should be available. If there are restrictions on publicly sharing data—e.g. participant privacy or use of data from a third party—those must be specified.

“Reviewer #1: Yes”

**Response**: Thank you for your positive evaluation regarding this criterion

**Comment 5**: “Is the manuscript presented in an intelligible fashion and written in standard English?

“PLOS ONE does not copyedit accepted manuscripts, so the language in submitted articles must be clear, correct, and unambiguous. Any typographical or grammatical errors should be corrected at revision, so please note any specific errors here.

“Reviewer #1: No”

**Responses**: We appreciate your feedback regarding the language quality of our manuscript. We recognized the language issues and copyedited the entire manuscript, revising it paragraph by paragraph to enhance clarity and ensure it meets the highest language standards. We hope these revisions address your concerns and improve the overall clarity and readability of our manuscript.

# **Review Comments to the Author**

**R1 Comment 1**: “General: The protocol has picked an indispensable public health gap that requires prompt intervention and follow up.”

**Response**: Thank you for your positive evaluation of our protocol.

**R1 Comment 2**: “Title: Very long and needs to be concise as “Improving the detection and management of common non-communicable diseases in adult population in Sidama National Regional State, Ethiopia: A study protocol”

**Responses**: We appreciate the suggestion. We accepted the recommended title with a minor modification. To signify the setting, we included the word “rural” in the title. The revised submission title is “**Improving the detection and management of common non-communicable diseases in adults in rural Sidama National Regional State, Ethiopia: Study protocol.**”

**R1 Comment 3:** “***Abstract***:

“In the background section it is read as “PEN in Ethiopia is at an early stage and the readiness of rural primary healthcare units (PHCUs) to implement the strategy is unknown. We, therefore, propose to apply the strategy in the catchment areas of DobeToga Health Center, a rural PHCU in Sidama National Regional State (SNRS), Ethiopia, and improve the NCDs care among adults aged≥45 years. “

“I think the authors are not well aware of the Ethiopian Healthcare clinical guidelines (EPHCG) adopted by the ministry of health. The detection and management of hypertension and DM type 2 is well addressed. The problem is insufficient implementation of this guideline in primary healthcare units probably including in Sidama region. That is where you authors need to emphasize on.

“I am also skeptical about the age group you are referring to (45 year and above) because there is age shift and younger persons are also acquiring the situation these days and the service should be rendered to all adults without segregation.

**Responses**: Thank you very much for this crucial comment. Following your suggestions, we reviewed the Ethiopian Primary Healthcare Clinical Guidelines (EPHCG) adopted by the Ministry of Health. We concurred with the reviewer's suggestions and have revised the manuscript accordingly. Specifically, we focused on the study area as reflected in lines 15-20 of the revised manuscript, changing from: “…However, the implementation of the PEN in **Ethiopia** is at an early stage, … ” to: “…However, the implementation of the PEN in **rural health facilities in Sidama National Regional State** is at an early stage….”

Regarding the age group our study is targeting, we recognize the increasing burden of DM type 2 and hypertension among younger individuals. However, for this study, the age group of 45 years and above was chosen due to financial and logistical constraints. We believe that focusing on this age group allows us to address a critical segment of the population at higher risk for the conditions. We will attempt to consider a broader age range to include younger individuals affected by these conditions in subsequent initiatives if resources allow. We appreciate your understanding of the constraints of this study and value your input for guiding potential future research directions.

**R1 Comment 4**: “Aim (in the Abstract): is too long and needs revision.

**Responses**: Thank you again for helping us improve our manuscript. We have revised the Aim (both in the Abstract (lines 22-26) and the body of the manuscript (lines 126-133)) to ensure it is more succinct while still capturing the essence of our study's objectives.

**R1 Comment 5:** “***Methods in the abstract*** are not narrated specifically in terms of tenses. Please distinguish what was already conducted and what will be conducted next.”

**Responses**: We appreciate the comment, which helps improve the readability of the manuscript. In response to your feedback, we have ensured that the Methods (both in the Abstract and the body of the manuscript) are narrated clearly in terms of tenses to distinguish between completed and ongoing/future activities. Specifically, we have made the following updates:

- Phase 1 activities: We completed the phase 1 activities and collected data from the community-based screening. Currently, we are in the process of cleaning the data obtained from these community-based surveys.
- Phase 2 activities: We are currently implementing the second phase of the study. In this phase, we are applying the care model, collecting follow-up data, and measuring biochemical and physiological parameters. This phase is expected to be concluded by February 2025.

In the revised submission, we have improved the Methods section in both the Abstract and the body of the manuscript. We have reported the completed activities in the past tense and ongoing/future activities in the present and future tenses.

We hope these revisions enhance the clarity and readability of our manuscript.

**R1 Comment 6**: “***Introduction***: There are very long sentences difficult to conceptualize in each paragraph. The efforts already in place by the Ministry of health to improve detection and management of NCDs have not been elucidated.”

**Responses**: Thank you for your valuable feedback and for highlighting the need for further clarification on the efforts already in place by the Ministry of Health to improve detection and management of NCDs.

We have carefully reviewed the manuscript and identified the long sentences that may pose challenges for readers. We have revised these sentences to improve clarity and readability. Specifically, we have broken down complex sentences into shorter ones. These changes have been made throughout the manuscript to enhance the overall flow and comprehension.

Furthermore, to elucidate the efforts undertaken by the Ministry of Health, we have incorporated the following information into the revised submission: “Ethiopia has been implementing various measures to address the growing burden of NCDs, such as hypertension and diabetes. These measures include developing a national guideline for early detection and treatment and enhancing the capacity of primary healthcare units (PHCUs) in low-resource settings [2,21–23]. The PHCUs are crucial for addressing NCDs and providing integrated, equitable care. Enhancing the diagnostic capacity for NCDs is one of the strategic initiatives in Ethiopia's current national strategy for preventing and controlling cardiovascular and chronic diseases [24].” (lines 94-100)

We hope these revisions improve the clarity of the manuscript and adequately highlight the efforts made by the Ministry of Health.

**R1 Comment 7: “Objectives**: There are redundant objectives like to determine the prevalence of undiagnosed diabetes etc…. (whatever the methods of detection are….)”

**Responses**: Thank you for your feedback. We have taken your comments into account and revised the objectives. We eliminated redundant objectives and kept the relevant ones. Consequently, we have reduced the number of objectives from 14 in the original submission to 8 in the revised manuscript. (lines 134-156)

**R1 Comment 8:** “There are no clear objectives stated for the qualitative study designs.”

**Responses**: Thank you for your feedback. We do not have a distinct objective that is addressed by the qualitative data. Instead, we have incorporated qualitative data to complement the quantitative data regarding the readiness of health facilities to implement the WHO PEN in rural Sidama, as outlined in the Methods section of the manuscript. We believe that including qualitative data will offer a deeper understanding of the challenges and facilitators involved in implementing this strategy.

**R1 Comment 9** “Willingness to pay for glycolated hemoglobin …..why was it a special interest for the investigators when the most difficult part is to pay for drugs which is to be used lifelong than the investigation done periodically.

**Responses**: We appreciate your concern regarding the emphasis on willingness to pay (WTP) for glycated hemoglobin (HbA1c) tests. Our focus on WTP for HbA1c testing stems from its critical role in diabetes management. Regular HbA1c testing is crucial for monitoring long-term blood sugar control, which greatly influences treatment strategies and outcomes. Understanding the financial feasibility and WTP for these tests can provide valuable insights for developing practical financial strategies for diagnosing diabetes.

We recognize that the cost of lifelong medication is a significant burden. However, medications for hypertension and diabetes are included in the Ethiopian Essential Health Services Package (EHSP) and the benefit packages of the Ethiopian community-based health insurance schemes. These actions help alleviate the burden of medication costs and make them more affordable.

In contrast, HbA1c tests are not prioritized as highly and are less likely to be included in the CBHI benefit package or the EHSP. Given the role of these tests in the effective management of diabetes, we believe it is crucial to assess the affordability of these diagnostics from the demand side. This is why we included this aspect in our study.

**R1 Comment 10:** “What about assessment and management of patients with multi-morbidity or co-morbidity?”

**Responses**: Thank you for the comment. Individuals with co-morbidity with hypertension and diabetes are included in the care model as the criteria to be part of the follow-up is diagnosis with type 2 DM, hypertension, or pre-DM.

**R1 Comment 11:** “Please focus on the key research questions as you are somewhat confused with many objectives…”

**Response**: We revised the research objectives and focused on the eight objectives in the updated submission. Thank you for the guidance.

**R1 Comment 12: “*Methods***:”

**Comment 12.1: “**Please clearly indicate the study setting…. Is it about the catchment area of a health center or more than that to conduct your study?”

**Responses**: We have updated the Setting section in the revised submission. Our study focuses on the four catchment kebeles of Dobe-Toga Health Center: Gonowa-Gabalo, Dobe-Toga, Howolso, and Gobe-Hebisha. These four kebeles have a total population of 38,874 people, of which 5,054 individuals are aged 45 and above. These individuals constitute our source population. We have tried to clearly reflect this information in the resubmission.

**Comment 12.2:** “It seems like you will be employing a mixed design…, Which mixed design, are you employing and why?”

**Responses**: Thank you for this question. For Objective 5, we utilized a concurrent qualitative and quantitative study design to assess the readiness of primary healthcare units in Sidama National Regional State to implement the WHO PEN. This concurrent design complemented the quantitative findings and enhanced our conclusions about the facilities' readiness. We have already collected both quantitative and qualitative data for this objective and are currently analyzing the data. The other objectives are being addressed using only quantitative data.

**Comment 12.3:** “Would you please indicate your study population (both for qualitative and quantitative)? And how to select your study participants (sampling procedure) at each level has to be displayed schematically”

**Responses**: Thank you for this comment. For the community-based cross-sectional studies, the source population included all adults aged 45 or older residing in the catchment areas of Dobe-Toga Health Center. The follow-up focuses on adults from this same population who were diagnosed with elevated blood pressure or diabetes mellitus during the cross-sectional survey. For the cross-sectional surveys of PHCUs, all health centers and primary hospitals in the SNRS were considered the source population. We have already described these in the Populations subsection in the Methods. The sampling procedure has already been displayed schematically in Fig 1 under the “Sampling procedure and follow-up schedule” sub-section of the Methods section (lines 319-344)

We did not include the population for the qualitative study in the initial submission. The population for the qualitative data supplementing the quantitative data for Objective 5 consisted of healthcare experts and NCD focals at the regional, zonal, district, and facility levels. we have updated the Populations sub-section to include this information (lines 204-206).

**Comment 12.4:** “The sample size allocated for different kebeles is not clear… .what was the reason for having distinct sample sizes for different kebeles? Why was the design effect considered to be 2? Why not 1.5???”

**Responses**: We appreciate your comment. As detailed in the "Sampling Procedure and Follow-Up Schedule" sub-section of the Methods section, the sample was allocated to the four kebeles in proportion to their population sizes (lines 322-325). The kebeles do not have distinct sample sizes; instead, the number of participants assigned to each kebele varies based on its population size.

Regarding the design effect, we decided to use a value of 2 instead of 1.5 for the following reasons**: 1) We expected a higher level of clustering and a stronger intra-class correlation among the villages. Therefore, to more adequately capture the variations among the participants, we believed it was appropriate to increase the design effect. 2) We considered a design effect of 2 to increase the sample size and enhance the statistical power of the study.**

**Comment 12.5:** “The language to be used for the interview sessions has not been clearly mentioned. The issue of using local languages translations (back and forward)”

**Responses**: Thank you for this important question. As described in the Quality Assurance sub-section of the Methods section (lines 497-516), the quantitative data collection tools were translated into the local language, Sidaamu Affoo. The data are being collected through face-to-face interviews using the interviewer-administered Sidaamu Affoo version of the questionnaire.

The interview guide for the in-depth interview was originally drafted in Amharic and administered in the same language. However, the data collectors were bilingual and were able to facilitate interviews in Amharic, Sidaamu Affoo, or a combination of the two, as preferred by the informants. We have updated the manuscript to include this information in the Methods section (lines 493-496).

**Comment 12.6: “**Operation definitions of key outcome variables are missing”

**Responses**: Thank you very much for this reminder. We have included “Operational definition” in the revised submission (lines 365-449)

**Comment 12.7:** “What is the outcome variable for the cohort study and how will it be measured and what will you be measuring as incidence and its predictors etc.????”

**Responses**: Thank you very much for the question. the outcome variables for the follow-up studies are the level of systolic and diastolic blood pressure, blood glucose levels, and levels of HbA1c. We simply compare the differences in these measures before and after the interventions (pre-post design) using t-test. We do not need measuring the incidence

**Comment 12.8:** “Are you going to compute Kaplan Meier, cox regression etc.???”

**Responses**: Survival analysis is not currently part of our plan. However, once data collection is complete, if it becomes apparent that Kaplan-Meier or Cox regression analysis is needed, we will consider incorporating these methods into our analysis.

**Comment 12.9:** “Improving patient ownership: Please try to capitalize on the concept of ‘person or patient centered care’ which is a contemporary concept to be dealt with as part of self-care and adherence to self-care.”

**Responses**: Thank you for your valuable suggestion. We have incorporated the concept of 'person or patient-centered care' into our activity to improve patient ownership in hypertension and diabetes management. we have also updated the manuscript to reflect this (lines 268-291).

**Comment 12.10: “**The 31 items for self-care need to be validated in Ethiopian context.. Please provide explanation on this. If not validated locally. It needs to be validated though you mentioned it as‘standardized’”

**Responses**: Thank you for your insightful comment. While H-SCALE tool have been standardized and used in several studies within Ethiopia, indeed, they have not undergone local validation. Validation within the Ethiopian context would enhance the accuracy and reliability of the tool by ensuring it is culturally and contextually appropriate.

However, due to resource constraints, we are not in a position to conduct a full validation study at this time. Instead, we rely on the existing body of research that has successfully utilized these items in similar settings within Ethiopia. Additionally, we conducted a pre-test and had experts proficient in the local culture translate the materials into the local language, which should enhance the validity of the tool. We will acknowledge any limitations this may introduce and address them transparently in the discussion section when we publish our findings.

**Comment 12.11: “**It seems that you don’t have a sample size for qualitative data…what will you be doing? And how will you do the analyses?”

**Response**: Thank you very much for this question. You are correct that we did not specify a sample size for the qualitative data in the original submission. The qualitative data for complementing Objective 5 were collected through in-depth interviews with purposively selected informants capable of providing rich and detailed information on the readiness of primary healthcare units to implement the WHO PEN strategy in the Sidama Region. These informants included regional, zonal, and district healthcare administrative experts, as well as NCD focal persons at selected administrative organs and health facilities. We conducted interviews until we reached information saturation, meaning no new themes or insights emerged. Accordingly, we conducted 10 in-depth interviews: one with a regional NCD focal person, one with a zonal health department expert, three with district health office NCD focal persons, and five with facility NCD focal persons at selected health centers and primary hospitals. In the revised manuscript, we have incorporated this information into the Sample Size Determination sub-section of the Methods section (lines 311-317).

For the analysis, we will use thematic analysis. This involves transcribing the interviews and coding the data to identify key themes and patterns. We will systematically review the transcripts to ensure that the coding is consistent and comprehensive. The identified themes will then be used to inform our understanding of the facilitators and barriers to implementing the WHO PEN strategy in rural health settings. We have updated the manuscript to include this information in the Methods section (lines 469-474)

**Comment 12.12: “**In your data analyses, what was the reason for employing R and stata softwares? Was that not possible to pick one preferable? R as it is the most robust or powerful one?”

**Response**: Thank you for your comment. Initially, we were unaware that R included a package to calculate the Krinsky and Robb mean/median WTP, which can be easily done in Stata using the WTPCIKR package. However, we have since discovered that the DCchoice package in R calculates the mean/median WTP. Consequently, we have decided to use only R for the statistical analysis and have updated the statistical analysis plan in the revised manuscript to reflect this change (lines 450-476).

**Comment 12.13: ”**Overall comment: Do you think that your study will yield a different result as there are many studies with larger scope than yours on NCDs assessment and management in Ethiopia?”

**Response**: While it is true that there are many studies with a larger scope on NCD assessment and management among communities in Ethiopia, our study focuses on specific aspects and unique contexts within the Sidama Region. Our methodology involves taking three measures of blood pressure in the community, followed by further confirmation at health centers. This approach provides a more accurate picture of the hypertension burden among the older rural population in Ethiopia compared to existing studies.

Additionally, to our knowledge, we are the first to use the HbA1c test for confirmation of hyperglycemia measured in fasting blood sugar (FBS), which will enhance the accuracy of diagnosing this condition. Therefore, we anticipate that our results will differ from those in the current literature.

Furthermore, we are examining various facets of public health that intersect with the NCDs care and management. This comprehensive approach aims to provide a holistic picture, which can better inform decision-making.
