# Supplementary material for: Improving the detection and management of common non-communicable diseases in adults in rural Sidama National Regional State, Ethiopia: Study protocol
Source: PLoS One. 2026 Apr 20;21(4):e0302667. doi: 10.1371/journal.pone.0302667 (PMC13094975; doi:10.1371/journal.pone.0302667)
Supplement: Attachment — Submitted filename: Response_to_Reviewers_auresp_2.docx [file pone.0302667.s002.docx]

**Responses to the editor and the reviewer**

**Manuscript ID:** PONE-D-24-13998R1

**Title:** Improving the detection and management of common non-communicable diseases in adults in rural Sidama National Regional State, Ethiopia: Study protocol

**Journal:** PLOS ONE

Dear Dr. Okungu and Reviewers,

We would like to thank you and the reviewers for the time and effort invested in reviewing our manuscript and for the constructive comments provided. We have carefully revised the manuscript to address all points raised. Changes made in the manuscript are highlighted in the tracked version submitted alongside this letter.

# **Reviewer’s comments and responses**

**Comment 1:** “As PEN approach is still in its early stage of implementation in your study setting, you may not be able to evaluate its performance. You may simply assess readiness of PHCU in detecting and managing common NCDs.”

**Response:** We thank the reviewer for this insightful comment. We would like to clarify that our study does not evaluate the existing WHO PEN program, which is indeed in its early stage in our setting. Instead, we developed and implemented a WHO PEN–based care model for hypertension and diabetes diagnosis and management in a rural setting. Our study evaluates the effectiveness of this locally implemented model, rather than the original WHO PEN framework. This distinction has been clarified in the revised manuscript.

**Changes made:**

- Abstract (lines 22–23): Revised to: “We primarily aim to evaluate the effectiveness of a WHO PEN–based care model, which we implemented in a rural setting…” (previously: “We aim to evaluate the WHO PEN–based care model's effectiveness in controlling blood pressure and glucose levels among older adults.”)
- Study Aims section (lines 129–130): Revised to: “The study primarily aims to evaluate the effectiveness of a WHO PEN–based care model, which we implemented in a rural setting…” (previously: “We aim to evaluate the WHO PEN–based care model's effectiveness in controlling blood pressure and glucose levels among older adults.”)

In addition, the intervention we implemented is described in detail in both the previous and current submissions in the Methods section (lines 219-294).

**Comment 2:** “What was the reason for picking hypertension and diabetes? Why not other NCDs?”

**Response:** We appreciate this crucial question. Hypertension and diabetes were selected due to their **high prevalence, public health importance, and feasibility of measurement** within our study resources. These conditions are also prioritized under national and global NCD strategies, enhancing the potential policy relevance of our findings.

**Changes made:** - Introduction section, line 111-115 now includes a rationale for focusing on hypertension and diabetes over other NCDs

**Comment 3**: “What was your outstanding reason to use qualitative method and how was the sample size computed or projected for the qualitative method? How were the data analysis done for qualitative method?”

**Response:** Thank you very much for this valuable comment. We have clarified that the qualitative component is included to triangulate the quantitative findings on readiness and to provide a richer understanding of PHCU readiness to implement the WHO-PEN. These were described in Methods section of the submission. The sample size for qualitative interviews was determined based on the principle of data saturation, a widely accepted approach in qualitative research (described in the Sample Size Determination section, lines 323–327). Qualitative data were analyzed using thematic analysis, which involved transcribing the interviews and manually coding the data to identify key themes and patterns (explained in the Statistical Analyses section, lines 486–491).

**Comment 4:** “Why are you referring to Dobe Toga while collecting your data from 41 health centers and 4 primary hospitals? This has to be corrected.”

**Response:** We appreciate the reviewer’s careful reading of our manuscript. We would like to clarify that the primary objective of our study is to implement and evaluate the effectiveness of a WHO PEN–based care model, which was specifically implemented in Dobe-Toga Health Center. This is the reason why Dobe-Toga is mentioned in the context of the intervention.

The reference to 41 health centers and 4 primary hospitals pertains to a different component of our protocol, namely the assessment of the readiness of primary healthcare units in Sidama to implement the WHO PEN. This assessment was conducted across multiple facilities, which is why both Dobe-Toga Health Center and the broader set of PHCUs are included in the manuscript.

For clarity, the study is structured into multiple objectives: The intervention study on WHO PEN effectiveness, undiagnosed hypertension, and T2DM was conducted in Dobe-Toga Health Center. The readiness assessment and related health system studies were conducted in the 41 health centers and 4 primary hospitals in Sidama.

**Comment 5:** “Is the data collection still ongoing in the study settings? If yes, you may have a different result. I think the data collection has to be completed before analyzing your findings and organizing a manuscript.”

**Response:** Thank you very much for your comment. We confirm that **data collection has been completed and currently, we are analyzing the data in accordance with the study objectives**. As this is a **study protocol**, no study findings have yet been analyzed. We have clarified that the manuscript describes **planned procedures and analysis**, with results to be reported after completion of data collection.

We hope that these revisions satisfactorily address all reviewer concerns. We are grateful for the constructive feedback, which has improved the clarity and rigor of our study protocol.

Thank you for considering our revised manuscript for publication in PLOS ONE.

Sincerely,

**Dr. Melaku Haile Likka**
