# Supplementary material for: Improving the detection and management of common non-communicable diseases in adults in rural Sidama National Regional State, Ethiopia: Study protocol
Source: PLoS One. 2026 Apr 20;21(4):e0302667. doi: 10.1371/journal.pone.0302667 (PMC13094975; doi:10.1371/journal.pone.0302667)
Supplement: Attachment — Submitted filename: Response_to_Reviewers_auresp_3.docx [file pone.0302667.s003.docx]

**Responses to the editor and the reviewer**

**Manuscript ID:** PONE-D-24-13998R2

**Title:** Improving the detection and management of common non-communicable diseases in adults in rural Sidama National Regional State, Ethiopia: Study protocol

**Journal:** PLOS ONE

**Dear Dr. Tope Michael Ipinnimo and Reviewer,**

We would like to thank you and the reviewer for the time and effort invested in reviewing our manuscript and for the constructive comments provided. We have carefully revised the manuscript to address all points raised. Changes made in the manuscript are highlighted in the tracked version submitted alongside this letter.

# **Editor’s comments and responses**

| **SN** | **Comments** | **Responses** |
| --- | --- | --- |
| 1. | “Journal Requirements: If the reviewer comments include a recommendation to cite specific previously published works, please review and evaluate these publications to determine whether they are relevant and should be cited. There is no requirement to cite these works unless the editor has indicated otherwise.” | We acknowledge this guidance. As no specific references were recommended by the reviewers, no additional citations were included. |
| 2. | “We recognise that the data collection for the study has now been completed and this has been a consequence of the time taken to review the manuscript. However, we require the protocol describing the study to be written in the future tense, as in the original version, so please can you re-write the text so that the manuscript describes the study that will be carried out (even if it has subsequently been completed). “Please note that if this is not undertaken appropriately then it may be necessary to reject your manuscript.” | Thank you for this guidance. We have carefully revised the manuscript to ensure that all descriptions of study procedures are written in the future tense, as required for a protocol paper, while retaining factual information and background in the past or present tense. This revision ensures that the manuscript now consistently describes the study as planned, in accordance with the journal’s requirements for protocol papers. We have shown the changes in track changes in the revised manuscript to facilitate your review |

# **Reviewer’s comments and responses**

| **SN** | **Comments** | **Responses** |
| --- | --- | --- |
| 1. | “Thank you for the detailed manuscript. The previous reviews have been attended to except the last comment which is still not very clear.” | We sincerely thank you for your time and valuable feedback, which has been instrumental in improving the quality of our manuscript and aligning it with the standards of the journal. We appreciate your acknowledgment that the previous comments have been addressed. Regarding the final comment, we have made specific revisions to clarify and respond to your concern, as reflected in lines 636–644 of the revised manuscript. The detailed modifications are also outlined in our response to Comment 3. |
| 2. | “The manuscript consistently employs past tense to describe completed procedures, such as “surveys were conducted” and “data were collected.” I think Protocols are expected to outline planned methods and future procedures. The use of past tense implies that the protocol was prepared after data collection which could be concerning.” | Thank you for this guidance. We have carefully revised the manuscript to ensure that all descriptions of study procedures are written in the future tense, in line with protocol reporting standards, while factual information and background are retained in the past or present tense as appropriate.  In the previous version, some procedures were described in the past tense because, due to delays in the journal review process, certain study activities were completed after the initial protocol submission but before the second revision of the manuscript. To avoid any ambiguity and to comply with protocol conventions, we have now consistently rewritten all methodological descriptions in the future tense to reflect the study as originally planned.  All revisions have been indicated using track changes in the revised manuscript to facilitate your review. |
| 3. | “The manuscript lacks an explanation for the completion of quantitative data collection prior to protocol submission, whereas qualitative data collection has not yet commenced. “I think clarification of the study timeline is necessary, along with an explanation of how protocol integrity was preserved when a portion of the study was conducted before protocol submission”. | Thank you for this important comment. One quantitative component of the study (Objective 5), which involved observation of health facility readiness to implement the WHO PEN intervention, was conducted prior to protocol submission for budgetary reasons related to the closure of a fiscal year. This activity was undertaken after ethical clearance had been obtained and was carried out strictly in accordance with the originally approved protocol, using predefined objectives, tools, and procedures. Objective 5 is the only study objective with a qualitative component; however, no qualitative data for this objective were collected at that time. Importantly, no data management, analysis, or interpretation was undertaken for the quantitative data collected for Objective 5 prior to protocol submission.  All remaining data for the other study objectives (Objectives 1–4 and 6–8), which do not include qualitative components, were not collected prior to protocol submission. In addition, the qualitative data for Objective 5 were also not collected prior to protocol submission.  To align with protocol reporting standards, we have revised the manuscript to describe all study procedures consistently in the future tense, presenting the protocol as originally planned. The early completion of one quantitative component did not result in any modification to the study design, objectives, data collection instruments, or analysis plans, thereby preserving full protocol integrity. |
| 4. | “Please can you specify the analytic approach for the qualitative data (such as thematic or content analysis), coding procedures and software that will be utilized.” | Thank you for this comment. We have revised the manuscript to provide a clearer and more detailed description of the qualitative data analysis approach, including the analytic method, coding procedures, and software to be used.  Specifically, we have expanded the Methods section (lines 636–644) to state that qualitative data will be analyzed using a thematic analysis approach. We now clarify that all interviews will be audio-recorded, transcribed verbatim, and imported into ATLAS.ti software for data management and analysis. We also describe the development of an initial coding framework informed by the study objectives and relevant literature, the systematic and iterative coding process to ensure consistency and comprehensiveness, and the grouping of related codes into categories and overarching themes. Finally, we specify how the resulting themes will be used to interpret facilitators and barriers to implementing the WHO PEN strategy in rural primary healthcare settings in the SNRS. |

We hope that these revisions satisfactorily address all the editor’s and reviewer’s concerns. We are grateful for the constructive feedback, which has improved the clarity and rigor of our study protocol.

Thank you for considering our revised manuscript for publication in PLOS ONE.

Sincerely,

**Dr. Melaku Haile Likka**
